# GABAergic regulation of *Locus coeruleus* activity in necdin-deficient mice, an animal model of Prader-Willi syndrome

**DOI:** 10.1186/s11689-025-09667-9

**Published:** 2025-12-30

**Authors:** Li-Ping Tsai, Hao Chan, Wei-Chen Hung, Ming-Yuan Min, Sin-Jhong Cheng, Chen-En Yang, Chun-Hsien Yu, Shi-Bing Wong

**Affiliations:** 1 Department of Pediatrics, Taipei Tzu Chi Hospital, Tzu Chi Medical Foundation, No. 289, Jiangguo Rd, Xindian Dist, New Taipei City, 23142 Taiwan; 2 School of Medicine, Tzu Chi University, No. 701, Sec 3, Jhongyang Rd, Hualien, 97071 Taiwan; 3 Department of Pediatrics, Heping Fuyou Branch, Taipei City Hospital, No.12, Fuzhou St., Zhongzheng Dist, Taipei, 100027 Taiwan; 4 Department of Life Science, College of Life Science, National Taiwan University, No. 1, Sec 4, Roosevelt Rd, Taipei, 10617 Taiwan; 5 Institute of Biomedical Science, Academia Sinica, 128 Section 2, Academia Road, Nankang, 11529 Taipei Taiwan

**Keywords:** Prader-Willi syndrome, Necdin, *Locus coeruleus*, GABA

## Abstract

**Background:**

Prader-Willi syndrome (PWS) is a neurodevelopmental disorder caused by loss of paternally expressed genes on chromosome 15q11–13, including *NDN*, which encodes necdin. Necdin deficiency has been linked to impaired visuospatial memory, social recognition, and stress regulation—features also seen in PWS. Previous work showed that necdin-deficient (Ndn + m/−p) mice exhibit reduced activity of noradrenergic neurons in the *locus coeruleus* (LC), a nucleus essential for arousal and stress responses. However, the mechanisms underlying LC hypoactivity remain unclear. Because GABAergic signaling is critical for LC excitability, this study examined the role of GABA_A_ ​and GABA_B_ ​receptor-mediated inhibition in Ndn + m/−p mice.

**Methods:**

Electrophysiological recordings from brainstem slices of wild-type (WT) and Ndn + m/−p mice were used to measure spontaneous firing rates (SFRs) of LC noradrenergic neurons. The effects of bicuculline (GABA_A_​ antagonist) and CGP54626 (GABA_B_​ antagonist) were tested. Whole-cell patch-clamp recordings assessed receptor-mediated currents. Western blotting quantified receptor subunit expression in peri-LC tissue. Immunocytochemistry and ELISA examined GABA_B_​ receptor expression and GABA release in cultured astrocytes.

**Results:**

LC-NE neurons in Ndn + m/−p mice exhibited significantly reduced baseline SFR compared with WT. Bicuculline did not alter firing in either genotype, whereas CGP54626 significantly increased SFR in WT but not in Ndn + m/−p neurons, indicating impaired GABA_B_ receptor-mediated tonic inhibition. Whole-cell patch-clamp experiments confirmed intact GABA_A_ receptor-mediated inward currents in both genotypes, while no GABA_B_ receptor-mediated phasic currents were detected. Western blot analysis revealed comparable expression of GABA_A_ receptor α2 subunit and GABA_B_R1 in peri-LC tissue between WT and Ndn + m/−p mice, suggesting functional rather than expression-level deficits. GFAP-positive cell density in the LC region was unchanged in vivo; however, in astrocyte cultures, Ndn + m/−p astrocytes exhibited greater proliferation by DIV 19 and consistently secreted higher levels of GABA, with significant elevations at later culture stages.

**Conclusion:**

Necdin deficiency selectively disrupts GABA_B_ receptor-mediated tonic inhibition of LC-NE neurons while preserving GABA_A_ receptor function. Elevated astrocytic proliferation and GABA release may further enhance ambient inhibition, contributing to LC hypoactivity and the neurobehavioral phenotypes of PWS. These findings identify GABA_B_ receptor dysfunction and astrocytic dysregulation as potential mechanistic targets for therapeutic intervention in PWS.

## Background

Prader-Willi Syndrome (PWS) is a complex genetic disorder arising from the loss of function of paternal genes located on chromosome 15q11-13 [1]. Patients with PWS exhibit a wide range of symptoms, including neonatal hypotonia, hyperphagia, obesity, cognitive impairments, and significant behavioral disturbances. Among the affected genes, the imprinted gene NDN, which encodes the multifunctional protein necdin, plays a critical role in neuronal differentiation, survival, and synaptic plasticity [2–4]. Loss of necdin function possibly contributes to several neuropsychiatric manifestations observed in PWS, including anxiety, temper outbursts, emotional dysregulation, and compulsive behaviors.

Recent studies on necdin-deficient mice, an animal model that mimics several neurobehavioral symptoms of PWS, have provided critical insights into the underlying mechanisms of the disorder [4, 5]. These mice exhibit functional abnormalities in the *locus coeruleus* (LC), a brainstem nucleus that serves as the primary source of norepinephrine (NE) in the central nervous system (CNS) [4]. The LC plays a central role in modulating arousal, attention, and stress responses [6], and its dysfunction has been implicated in several neuropsychiatric conditions relevant to PWS. In necdin-deficient mice, the spontaneous firing rate (SFR) of LC-NE neurons is significantly reduced, suggesting an overall hypoactive arousal system that may contribute to behavioral abnormalitie [4]. The activity of LC neurons is tightly regulated by inhibitory GABAergic inputs, which help maintain homeostatic neuronal excitability and prevent hyperactivity [7–10]. While GABAergic deficits have been reported in the forebrain of necdin-deficient mice [11], the status of GABAergic signaling specifically within the peri-LC region remains unclear.

Gamma-aminobutyric acid (GABA) is the major inhibitory neurotransmitter in the CNS and exerts its effects through two main receptor classes: GABA_A_ and GABA_B_ receptors. GABA_A_ receptors (GABA_A_Rs) are ionotropic chloride channels that mediate fast synaptic inhibition and contribute to both phasic and tonic inhibitory control [12]. In contrast, GABA_B_ receptors (GABA_B_Rs) are G protein-coupled receptors (GPCRs) that mediate slower, longer-lasting inhibitory effects through modulation of intracellular second messenger cascades, including suppression of calcium influx and activation of potassium currents [13, 14]. A key distinction lies in their temporal kinetics: GABA_A_Rs mediate rapid hyperpolarization through chloride conductance, while GABA_B_Rs exert sustained inhibition via downstream signaling pathways. Dysfunction in GABA_A_Rs has been associated with epilepsy, anxiety disorders, and schizophrenia [15–17], whereas abnormalities in GABA_B_Rs signaling have been linked to depression [18], addiction [19], and cognitive and behavioral phenotypes in autism spectrum disorder and fragile X syndrome [20]. 

In patients with PWS, neuropsychiatric symptoms such as anxiety, mood instability, and compulsive behaviors are prevalent, suggesting a possible role for GABAergic dysfunction—particularly involving the GABA_B_ receptor system—in the pathogenesis of these features. GABA_B_Rs expressed on LC neurons have been shown to exert tonic inhibitory control over LC-NE activity [21]. Disruption of this regulatory mechanism could contribute to core PWS symptoms, including impaired stress responsiveness and compulsive behaviors. While previous studies have identified GABAergic abnormalities in forebrain regions of Ndn + m/−p mice [11], the role of GABA_A_ and GABA_B_ receptor systems in regulating LC function remain to be fully characterized.

To address this gap, the present study investigates GABAergic regulation of LC-NE neurons in necdin-deficient mice. We examine GABA receptor expression, inhibitory synaptic transmission, and neuronal excitability in the LC. Our findings aim to clarify the contribution of region-specific GABAergic dysfunction to noradrenergic hypoactivity and neurobehavioral abnormalities in PWS, and may provide insights into potential therapeutic targets for modulating arousal and emotional regulation in this disorder.

## Materials and methods

### Animals

Heterozygous necdin-deficient mice (B6.Cg-Ndn^tm1ky^) were purchased from RIKEN (Saitama, Japan). *Ndn* was maternally imprinted, and only the paternal allele was functional. Therefore, heterozygous males carrying an NDN-deleted allele were bred from wild-type (WT) females (ICR background) to generate WT (+ m/+p) and heterozygote (+ m/−p) animals in which the paternal allele was deleted, resulting in necdin deficiency. Only male animals were used in the experiments. The use of animals was approved by the Ethical Committee for Animal Research of Taipei Tzu Chi Hospital (110-IACUC-024), and the study was conducted in accordance with the National Institutes of Health guidelines. Every effort was made to minimize the number of animals used and their suffering.

### Western blotting

Peri-LC tissue was microdissected from sagittal brainstem sections under a dissecting microscope. LC neurons, which are relatively large and distinguishable at low magnification, were used as anatomical landmarks to guide precise resection of the peri-LC region (Fig. 3A). Total protein content was evaluated using a Bradford assay and results were used to load the same amount of protein per lane on a 10% Tris-HCl gel. After electrophoresis, proteins were transferred to a nitrocellulose membrane and then incubated overnight at 4˚C with the primary antibody against GABA_A_ (abcam ab72445, Cambridge, UK) or GABA_B_ (abcam ab55051, Cambridge, UK) receptor. The membrane was incubated for 1 h with the secondary antibody, horseradish peroxidase-conjugated 1 : 5000, and developed after treatment with ECL plus. The membrane was exposed to a radiographic film and imaged/quantified on imaging system. The same procedure was used for beta-actin immunostaining as control. Signal intensities for each lane were normalized to beta-actin signal for that lane.

### Electrophysiology

Brainstem slices (300 μm thick) were prepared using a vibratome (Leica VT1000 S, Leica Biosystems, Nussloch, Germany) and transferred to a recording chamber mounted on an upright microscope (BX51WI, Olympus Optical Co., Tokyo, Japan). Slices were continuously perfused with artificial cerebrospinal fluid (ACSF) at a rate of 2–2.5 mL/min, bubbled with a gas mixture of 95% O₂ and 5% CO₂. The ACSF composition was as follows (in mM): 119 NaCl, 2.5 KCl, 1 NaH₂PO₄, 1.3 MgSO₄, 2.5 CaCl₂, 26.2 NaHCO₃, and 11 glucose, with a pH adjusted to 7.4 and osmolality maintained between 290 and 300 mOsm. Neurons were visualized using a digital camera system (C10600 ORCA-R2, Hamamatsu, Japan). Patch pipettes were fabricated from borosilicate glass tubing (G150F-4/GC150F-10; outer diameter: 1.5 mm, wall thickness: 0.32 mm; Warner Instruments, Hamden, CT, USA), yielding a tip resistance of 6–8 MΩ when filled with intracellular solution. Electrophysiological recordings were performed using a Multiclamp 700B amplifier (Molecular Devices, Sunnyvale, CA, USA). Signals were low-pass filtered at 2 kHz and digitized at 10 kHz using a Micro1401 interface and Spike2 software (Cambridge Electronic Design, Cambridge, UK). For whole-cell current-clamp recordings, the pipette solution contained (in mM): 131 K-gluconate, 20 KCl, 10 HEPES, 2 EGTA, 8 NaCl, 2 ATP, and 0.3 GTP, with pH adjusted to 7.2–7.3 and osmolality to 300–305 mOsm. Recordings were included only if the resting membrane potential was at least − 40 mV (without holding current) and action potentials could overshoot 0 mV. For voltage-clamp recordings, neurons were held at − 70 mV unless otherwise indicated. In cell-attached configuration, spontaneous action potential firing was recorded at 33 °C. For these recordings, the pipette was filled with ACSF [22, 23]. Cells with firing rate greater than 2 standard deviations above or below the group mean were considered as outliers and were excluded from analysis.

### Primary astrocyte culture

Two-day-old mice were anesthetized with isoflurane and decapitated. Brainstem tissues were rapidly dissected and placed in ice-cold Hank’s Balanced Salt Solution (HBSS; Gibco), pH 7.4. After removal of meningeal and vascular tissue, samples were washed three times with cold HBSS. The brainstem tissue was then minced into small fragments and enzymatically dissociated in 1 mL of 0.25% trypsin-EDTA (Gibco) at 37 °C for 15 min. Enzymatic digestion was terminated by the addition of 1 mL of fetal bovine serum (FBS; Peak), and the cell suspension was gently triturated using a pipette to ensure single-cell dispersion. Dissociated cells were seeded onto 100 mm tissue culture dishes pre-coated with poly-D-lysine and maintained in DMEM/F12 medium (Gibco) supplemented with 10% FBS, 15 mM NaHCO₃ (Wako), and 10 µg/mL penicillin-streptomycin (Gibco). Cultures were incubated at 37 °C in a humidified atmosphere containing 5% CO₂. The culture medium was replaced every 2 days to maintain optimal growth conditions for astrocyte proliferation.

### Immunohistochemical staining

#### IHC of LC-NE neurons and astrocytes

For fluorescent IHC staining following electrophysiology experiments, brain slices were fixed with 4% paraformaldehyde (PFA) dissolved in 0.1 M phosphate buffer (PB). The slices and cells were washed three times for 5 min each with 0.1 M PB, then incubated for 90 min in blocking solution (2% bovine serum albumin in PBST; 0.01 M phosphate-buffered saline with 0.3% Triton X-100). Slices were then transferred to the primary antibody solution: rabbit anti-tyrosine hydroxylase (TH) (1:1000; AB152, Merck Millipore, Burlington, Massachusetts, USA) and mouse anti-Glial Fibrillary Acidic Protein (GFAP) (1:1,000; MAB360, Merck Millipore) in PBST, and incubated overnight at 4 °C with gentle agitation. The following day, the slices were washed again with 0.1 M PB three times for 5 min each and then incubated for 2 h in a secondary antibody mixture containing goat anti-rabbit Alexa Fluor 594 and streptavidin-conjugated Alexa Fluor 488 (1:200; Jackson ImmunoResearch, West Grove, Pennsylvania, USA). Finally, the slices were washed with 0.1 M PB, mounted using Rapiclear mounting solution (SunJin Lab, HsinChu, Taiwan), and imaged with a Leica TCS SP8 Confocal Microscope (Leica Microsystems, Wetzlar, Germany). To quantify astrocyte numbers around the LC, unilateral confocal images were obtained at the rostral LC level (− 5.4 mm from Bregma). Astrocytes were counted within a 0.6 mm² square region of interest centered on the LC. Astrocytes were defined as DAPI-labeled nuclei surrounded by or overlapping with GFAP.

#### IHC of cultured astrocytes

Primary astrocytes were cultured from the brainstem region of 2-day-old wild-type and Ndn + m/-p mice as previously described. Astrocyte cultures were maintained for 7–19 days in vitro (DIV) before immunohistochemical staining. Cells were first washed twice with PBS and then fixed with 4% PFA in PBS for 15 min at room temperature. After fixation, cells were washed three times with PBS and then incubated for 90 min in blocking solution (2% bovine serum albumin in PBST; 0.01 M phosphate-buffered saline with 0.3% Triton X-100). The astrocyte cultures were then incubated overnight at 4 °C with primary antibodies diluted in blocking buffer: anti-GABA_B_ receptor 1 (1:500; abcam ab55051, Cambridge, UK), and anti-glial fibrillary acidic protein (GFAP, 1:1000; Santa Cruz sc-33673, Dallas, Texas, USA) to label astrocytes. After primary antibody incubation, cells were washed three times with PBS and incubated with appropriate secondary antibodies conjugated to Alexa Fluor dyes (Alexa Fluor 488 (1:200; Jackson ImmunoResearch, West Grove, Pennsylvania, USA) for 1 h at room temperature in the dark. Following secondary antibody staining, cells were washed and counterstained with 4’,6-diamidino-2-phenylindole (DAPI, 1 µg/mL in PBS) for 5 min to visualize cell nuclei. After final washes, coverslips were mounted using an anti-fade mounting medium. Fluorescent images were captured using a Leica TCS SP8 Confocal Microscope (Leica Microsystems, Wetzlar, Germany) under identical settings for all experimental groups.

### GABA measurement

Culture medium from primary astrocyte culture was collected at DIV 7, 13, 19. Enzyme-linked immunosorbent assay kits were used to measure GABA (Mybiosource MBS260709, San Diego, USA) according to manufacturer protocols. The astrocyte numbers were assessed using a hemocytometer manually.

#### Data and statistical analyses

Statistical analyses were performed using SPSS version 19.0 for Windows (IBM, Chicago, IL, USA). All data are presented as means and standard error of the mean. To assess the significance of independent variables in WT and Ndn + m/−p mice, independent t test was performed. Paired *t*-tests were used to compare the variables before and after drug application. The significance level was set at *p* < 0.05.

## Results

### Comparable GABA_A_ but impaired GABA_B_ responses of LC NE neurons of Ndn + m/-p mice

**Fig. 1:**
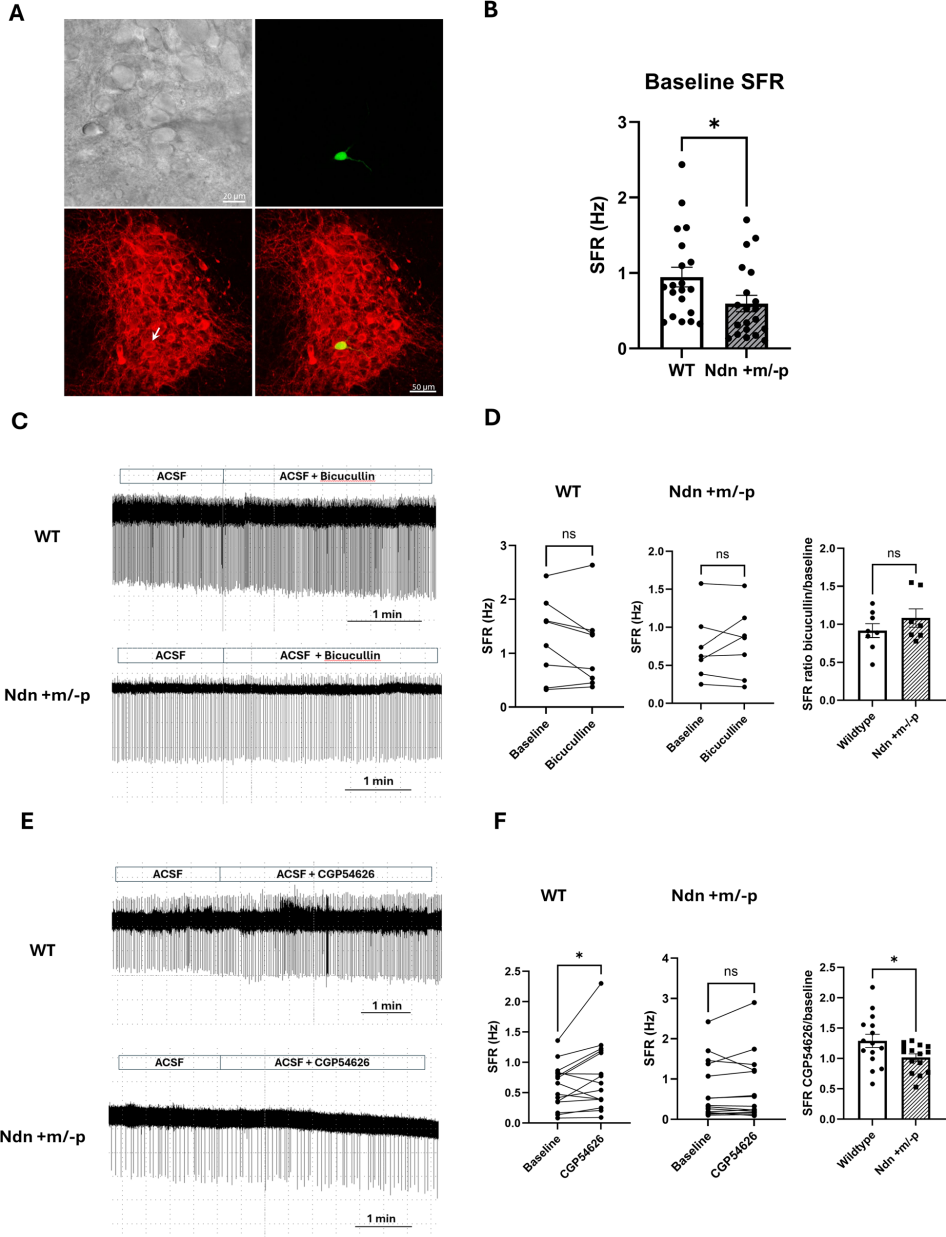
Differential Effects of GABA Receptor Antagonists on Spontaneous Firing Rates of LC Noradrenergic Neurons in WT and Ndn + m/-p Mice. **A** Representative confocal image showing the recorded LC neuron filled with biocytin (green) and immunopositive for tyrosine hydroxylase (TH; red), confirming its identity as an LC-NE neuron. **B** Comparison of baseline spontaneous firing rates (SFRs) of LC-NE neurons in WT (*n* = 23 from 15 mice) and Ndn + m/-p mice (*n* = 22 from 15 mice), showing significantly reduced SFR in Ndn + m/-p neurons. **C** Example traces of LC-NE neuron activity before and after treatment with bicuculline (GABA_A_ receptor antagonist). **D** Summary data showing no significant change in SFR after bicuculline treatment in either genotype (WT: *n* = 8; Ndn + m/-p: *n* = 7). **E** Example traces of LC-NE neuron activity before and after treatment with CGP54626 (GABA_B_ receptor antagonist). **F** Quantification showing that CGP54626 significantly increased SFR in WT mice (*n* = 15 neurons from 9 mice), but not in Ndn + m/-p mice (*n* = 15 neurons from 9 mice) Data are presented as mean ± SEM. *p < 0.05, independent t test

Firstly, we investigated the effects of bicuculline, a GABA_A_ receptor antagonist, and CGP54626, a GABA_B_ receptor antagonist, on the SFR of LC NE neurons. At high magnification, numerous large neurons could be identified for recording, and the recording cells (biocytin-positive) were also immunoreactive with an anti-TH antibody, documenting these as LC-NE neurons (Fig. 1A). In this experiment, 23 LC-NE neurons from 15 WT were and 22 LC-NE neurons from 15 Ndn + m/-p mice were recorded with cell-attached method, similar to our previous study [4]. Consistent with previous findings [4], the SFR of LC-NE neurons in Ndn + m/-p mice was significantly lower than in their wildtype counterparts ( *p* = 0.045, independent t test, Fig. 1B ). Eight LC-NE neurons of wildtype mice and 7 LC-NE neurons of Ndn + m/-p mice were treated with bicuculline, and the treatment did not alter the SFR in either WT or Ndn + m/-p mice (Fig. 1C and D). The SFR ratio after bicucullin treatment compared to baseline were 0.92 ± 0.09 and 1.08 ± 0.12 of wildtype and Ndn + m/-p mice, respectively (*p* = 0.285, independent t test, Fig. 1D), suggesting that GABA_A_ receptors play a minimal role in regulating the SFR of these neurons. However, CGP54626 treatment significantly increased the SFR in WT mice from 0.61 Hz to 0.78 Hz (*n* = 15 in 9 animals, Fig. 1E and F), while no significant change was observed in Ndn + m/-p mice (*n* = 15 in 9 animals, Fig. 1E and F). The SFR ratio after treating with CGP54626 over baseline of WT and Ndn + m/-p mice were 1.29 ± 0.42 and 1.02 ± 0.23, respectively (*p* = 0.037, independent t test, Fig. 1F). These results indicate that tonic inhibition via GABA_B_ receptors is crucial in modulating the SFR of LC NE neurons, and that Ndn + m/-p mice may have a deficiency in GABA_B_ receptor-mediated regulation.

### GABA_A_ and GABA_B_ currents of LC-NE neurons

**Fig. 2:**
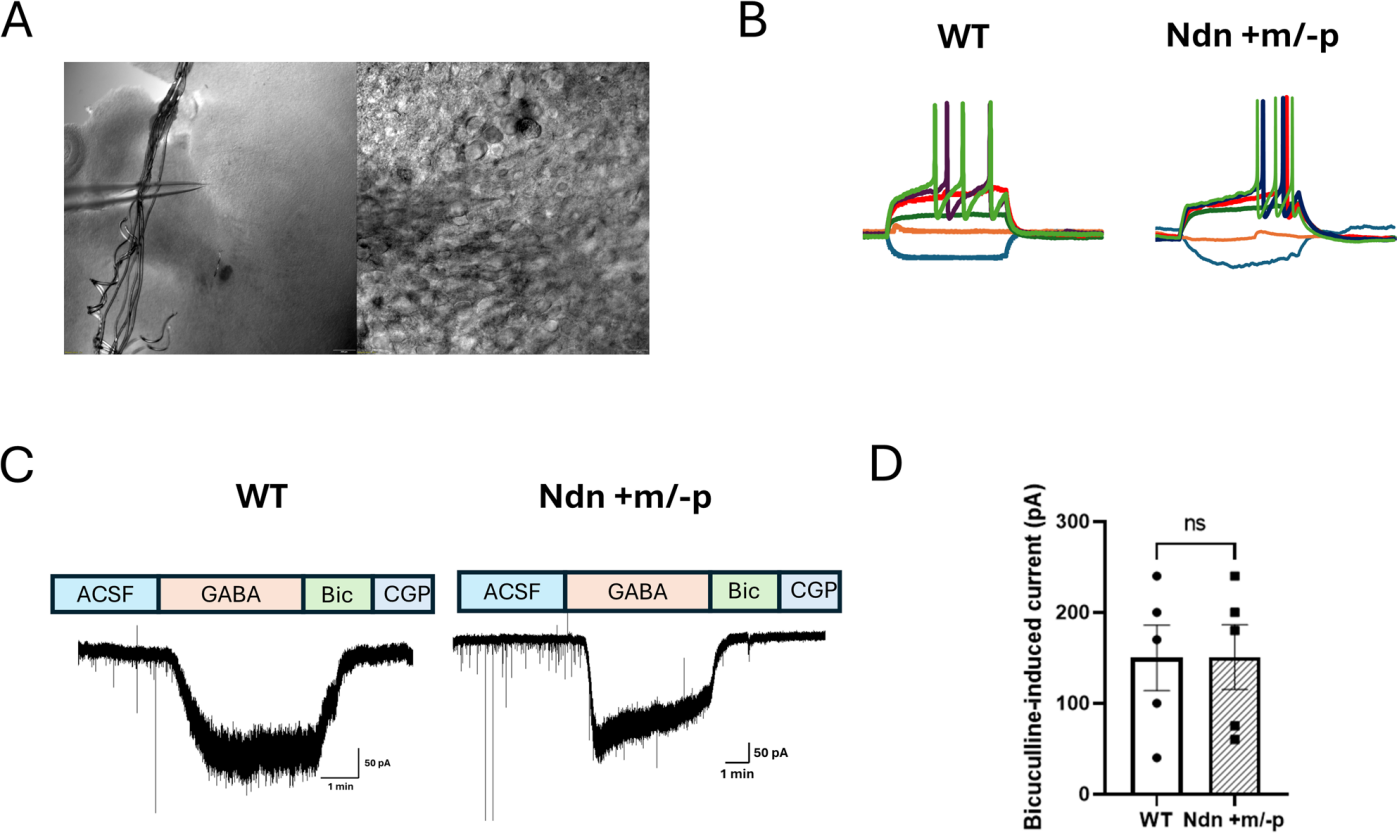
GABA Receptor Currents in LC-NE Neurons. **A** Low- and high-magnification images showing brainstem slice regions containing *locus coeruleus* (LC) neurons. **B** Representative current-clamp recordings from LC neurons in WT and Ndn +m/−p mice showing delayed onset of action potentials in necdin-deficient neurons following depolarizing current injection. **C** Representative traces of GABA receptor-mediated currents in voltage-clamp mode: application of bicuculline (20 µM) evoked inward currents in both WT and Ndn +m/−p LC neurons, while CGP54626 (GABAB receptor antagonist) failed to elicit measurable responses in either genotype. **D** Quantification of GABAA receptor-mediated currents shows no significant difference in current amplitude between WT (150 ± 35.8 pA) and Ndn +m/−p (151 ± 35.5 pA) neurons (n = 5 per group; p > 0.05)

To further assess receptor-specific inhibitory signaling, we recorded GABA receptor-mediated currents in LC-NE neurons using whole-cell patch-clamp techniques, as described in our previous studies [4, 8, 9]. To isolate GABAergic currents, the external bath solution was supplemented with synaptic blockers—5 mM kynurenic acid and 1 µM strychnine—to inhibit glutamatergic and glycinergic transmission, respectively. Consistent with prior findings [4], LC neurons from Ndn + m/−p mice exhibited delayed onset of action potential firing following depolarizing current injection, relative to WT controls (Fig. 2B). Under voltage-clamp conditions held at − 70 mV, local application of GABA (200 µM) evoked robust inward currents, which were completely abolished by the GABA_A_ receptor antagonist bicuculline (20 µM), confirming that the observed responses were mediated by GABA_A_Rs activation (Fig. 2C). In contrast, application of the GABA_B_R antagonist CGP54626 did not induce any detectable current in LC-NE neurons of either genotype, suggesting a minimal contribution of GABA_B_Rs to phasic synaptic activity under these recording conditions. Quantitative analysis of GABA_A_​R-mediated current amplitudes revealed no significant difference between genotypes, with peak currents measured at 150 ± 35.8 pA in WT and 151 ± 35.5 pA in Ndn + m/−p mice (*n* = 5 per group) (Fig. 2C and D). These findings indicate that phasic GABA_A_ ​R function is preserved in necdin-deficient mice and plays a predominant role in shaping LC-NE synaptic inhibition, while GABA_B_​R-mediated phasic responses are undetectable under basal conditions.

### Western blot analysis of GABA_A_and GABA_B_receptor expression in Peri-LC regions of WT and Ndn + m/-p mice

**Fig. 3:**
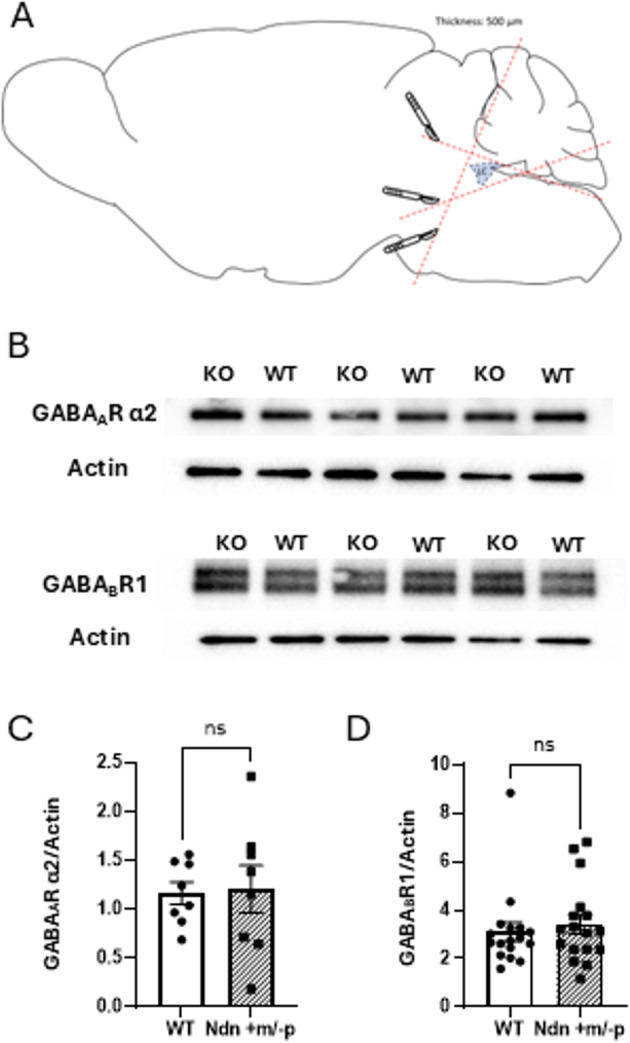
Western Blot Analysis of GABA_A_ and GABA_B_ Receptor Expression in Peri-LC Regions of WT and Ndn + m/-p Mice. **A** Schematic sagittal brainstem section illustrating the LC and surrounding peri-LC region dissected under a stereomicroscope. LC neurons, identified by their large soma size, were used as landmarks to guide tissue resection. **B** Representative Western blot images showing the expression of the GABA_A_R α2 subunit and actin in peri-LC tissue from WT and Ndn + m/-p mice. **B** Representative Western blot images showing the expression of GABA_B_R1 and actin in peri-LC samples across genotypes. **C** Quantification of GABA_A_R α2 subunit levels normalized to actin. **D** Quantification of GABA_B_R1 levels normalized to actin; data are presented as mean ± SEM

To investigate the molecular basis underlying the impaired GABA_B_R-mediated regulation of LC neuron activity in Ndn + m/−p mice, we next examined the expression levels of GABA_A_​ and GABA_B_​ receptor subunits in the peri-LC region using Western blot analysis. The peri-LC tissue was microdissected from sagittal brainstem sections under a dissecting microscope, using the anatomical location beneath the fourth ventricle and the higher transparency conferred by the large soma size of LC neurons as landmarks to identify the LC region (Fig. 3A). GABA_A_​Rs are heteropentameric chloride-permeable ion channels composed of diverse subunits from seven subfamilies (α1–6, β1–3, γ1–3, δ, π, θ, and ε) [24]. Among these, α2-containing GABA_A_​Rs play a key role in mediating fast synaptic inhibition in various central nervous system regions [25]. GABA_B_​Rs), in contrast, are metabotropic GPCRs that function as obligate heterodimers composed of GABA_B_R1 and GABA_B_​R2 subunits [26].

As shown in Fig. 3B and C, the expression of the GABA_A_​R α2 subunit was similar between WT and Ndn + m/−p mice. Quantitative densitometry analysis normalized to β-actin confirmed no significant difference in α2 subunit levels (WT: 1.2 ± 0.3; Ndn + m/−p: 1.2 ± 0.7; *p* = 0.886, *n* = 8 in each groups), indicating that necdin deficiency does not alter GABA_A_​R α2 expression in the peri-LC region. To clarify whether GABA_B_R1 expression was altered, we expanded the dataset to include 17 WT and 17 Ndn + m/−p mice. The normalized GABA_B_R1/β-actin ratio was 3.4 ± 1.6 in WT and 3.1 ± 1.6 in Ndn + m/−p mice, with no significant difference (*p* = 0.586; Fig. 3B and D). These findings demonstrate that GABA_B_R1 expression in the peri-LC is not significantly affected by necdin deficiency, suggesting that the impaired GABA_B_-mediated inhibition observed electrophysiologically reflects functional rather than protein-level changes.

### Excessive GABA secretion of primary astrocyte culture of Ndn + m/-p mice

Astrocytes, the most abundant glial cell type in the CNS, are critical regulators of neuronal function. Beyond providing metabolic and structural support, astrocytes directly influence neuronal excitability and synaptic transmission by expressing neurotransmitter receptors and releasing gliotransmitters, including GABA [27]. A previous study from a Japanese group using the same necdin-deficient mouse model demonstrated that necdin suppresses astrocyte differentiation via inhibition of the epidermal growth factor receptor (EGFR)/ERK signaling pathway, suggesting that loss of necdin may lead to abnormal astrocytic proliferation or maturation [28]. In parallel, our earlier work revealed that GABA_B_ ​receptors in LC neurons recruit ERK1 signaling to stabilize GABA_B_​ receptor-mediated currents and sustain tonic inhibition of neuronal firing [9]. Together, these findings suggest a potential mechanistic link: in necdin-deficient mice, aberrant astrocytic differentiation and function may enhance extracellular GABA levels, while impaired GABA_B_ receptor activity could diminish neuronal responsiveness to this ambient GABA.

**Fig. 4:**
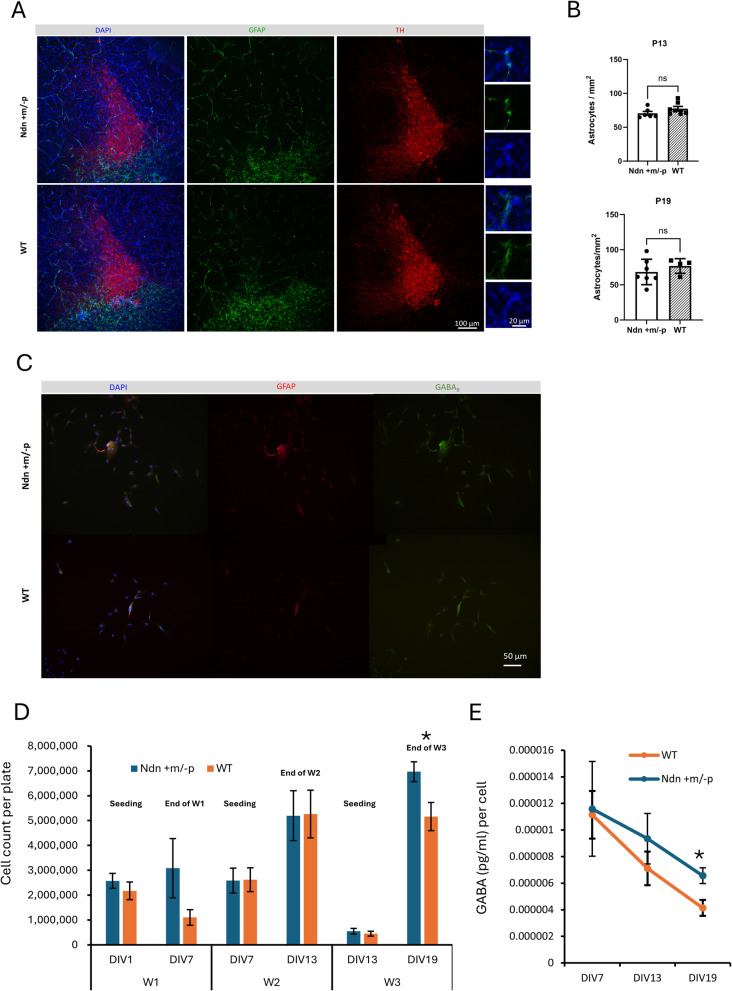
Astrocyte morphology, proliferation, and GABA secretion in WT and Ndn + m/−p mice. **A** Representative confocal image of GFAP-positive astrocytes in the peri-LC region of P19 mice. **B** Quantification of GFAP-positive astrocyte numbers in the peri-LC region of WT and Ndn + m/−p mice, showing no significant differences between genotypes. **C** Representative immunofluorescence staining of cultured astrocytes from the pons region at DIV19 of WT and Ndn + m/-p mice. Astrocytes were labeled with GABA_B_ receptors (green), glial fibrillary acidic protein (GFAP, red) to identify astrocytes, and DAPI (blue) for nuclear staining. **D** Proliferation of primary astrocytes across 3 weeks in vitro. Cell numbers were quantified at DIV1 (seeding), DIV7, DIV13, and DIV19 for independent cultures (W1–W3). Necdin-deficient astrocytes displayed increase in proliferation by DIV19. **E** GABA secretion from astrocyte cultures at DIV7, DIV13, and DIV19, normalized per cell. Ndn + m/−p astrocytes secreted significantly higher GABA than WT astrocytes at DIV19. All data are presented as mean ± SEM. *p < 0.05

To test this hypothesis, we first examined astrocyte morphology and density in the LC region of postnatal day 13 (P13) and P19 mice. Immunohistochemistry revealed that the number and overall morphology of GFAP-positive astrocytes in the peri-LC were comparable between WT and Ndn + m/−p mice at both stages (Fig. 4A and B), indicating that necdin deficiency does not significantly affect astrocytic density or gross morphology in vivo. To further evaluate whether necdin deficiency alters astrocyte growth dynamics, we quantified total cell numbers in primary astrocyte cultures over a 3-week period in vitro (DIV 0–19). Cultured astrocytes from the pons region were immunostained for GABABR1 (green), GFAP (red), and DAPI (blue) to visualize nuclei (Fig. 4C). At DIV 7 and DIV 13, cell numbers were comparable between WT and Ndn + m/−p cultures. By DIV 19, Ndn + m/-p astrocytes reached a higher mean cell count than WT (6.97 × 10^6^ vs. 5.16 × 10^6^, *p* = 0.019; Fig. 4D), indicating a modest but consistent increase in proliferation. In parallel, GABA concentrations in the culture medium were measured at DIV 7, 13, and 19. While GABA secretion per cell declined over time in both genotypes, Ndn + m/−p astrocytes consistently released higher levels of GABA than WT astrocytes, with a significant difference at DIV 19 (6.56 × 10^− 6^ vs. 4.14 × 10^− 6^, *p* = 0.019, Fig. 4E). Together, these findings suggest that necdin deficiency enhances astrocytic growth at later stages and alters GABA release dynamics, potentially elevating ambient GABA and contributing to excessive tonic inhibition in the LC microenvironment.

## Discussion

Our study demonstrates that necdin deficiency, a core molecular feature of PWS, leads to selective impairment of GABA_B_R-mediated tonic inhibition in LC-NE neurons, and increased astrocyte proliferation and GABA secretion in vitro. Because paternal allele of the NDN gene is silenced due to imprinting in PWS patients, both neuronal and non-neuronal cells, such as astrocytes and other glia, lack functional necdin protein. Using electrophysiological recordings, we showed that the GABA_B_R antagonist CGP54626 significantly increased the SFR of LC-NE neurons in WT mice but failed in necdin-deficient mice, indicating a functional loss of GABA_B_R-mediated regulation. In contrast, GABA_A_R function was preserved, as demonstrated by unchanged SFR following bicuculline application, intact GABA_A_R-mediated inward currents, and comparable α2 subunit expression between genotypes. Western blotting of an expanded cohort revealed no significant difference in GABA_B_R1 protein expression between WT and Ndn + m/−p mice, suggesting that the observed dysfunction reflects impaired receptor signaling rather than altered expression levels. Furthermore, astrocyte analyses revealed that while GFAP-positive astrocyte density and morphology in the LC were comparable between groups in vivo, Ndn + m/−p astrocytes in culture exhibited enhanced proliferation and elevated GABA release at later stages. These findings support the idea that necdin deficiency disrupts tonic inhibitory balance in the LC through a dual mechanism: functional impairment of neuronal GABA_B_R signaling and altered astrocytic regulation of ambient GABA levels.

The LC plays a central role in regulating arousal, attention, and stress responses as the principal source of NE in the brain. Both hypoactivity and hyperactivity of LC neurons are associated with neuropsychiatric dysfunction—ranging from cognitive impairment and poor alertness to anxiety and impulsivity​ [29]. GABA_B_ ​Rs expressed at peri- and extrasynaptic sites on LC neurons serve as key mediators of tonic inhibition by responding to ambient GABA levels [9, 21]. Elevated ambient GABA enhances GABA_B_​-mediated currents and silences LC-NE activity, a mechanism that can be pharmacologically mimicked by agonists such as baclofen [30]. In line with this, we found that CGP54626 significantly increased SFR in wild-type mice, consistent with the removal of tonic GABA_B_-mediated inhibition. This effect was absent in Ndn + m/−p mice, providing direct physiological evidence of dysfunctional GABA_B_​R signaling in the LC of a PWS model. Such LC hypoactivity may underlie the attention deficits, emotional dysregulation, and compulsive behaviors frequently observed in individuals with PWS.

Emerging evidence suggests that GABA_B_​R dysfunction also plays a role in neurodevelopmental disorders such as attention-deficit/hyperactivity disorder (ADHD), autism spectrum disorder, and intellectual disability. While ADHD is traditionally associated with dopaminergic and noradrenergic dysregulation, impaired GABAergic signaling—particularly involving GABA_B_​Rs—has also been implicated [31]. In mouse models, deletion of the GABA_B_​R1 subunit results in hyperactivity and learning deficits [32]. Moreover, mutations in GABBR2 (encoding the GABA_B_​R2 subunit) have been associated with autistic traits, cognitive impairment, and emotional lability, including temper tantrums and irritability—features highly reminiscent of the behavioral phenotype in PWS [33]. These converging findings suggest that GABA_B_​R disruption may contribute to intellectual disability and behavioral dysregulation through mechanisms such as impaired synaptic plasticity, excitatory/inhibitory imbalance, and aberrant neurodevelopment [32–34]. This study revealed that necdin-deficient mice, an animal model of PWS, presented selective GABA_B_ dysfunction. Our previous study using Ndn + m/−p mice further supports this view, demonstrating deficits in spatial memory, impaired social interaction, and altered sexual recognition—behaviors consistent with GABA_B_​R-linked dysfunction [5]. Together, these results underscore necdin’s critical role in GABA_B_R regulation and its contribution to core neuropsychiatric symptoms in PWS.

GABAergic abnormalities in PWS are supported by genetic and neuroimaging data. The 15q11–13 chromosomal region deleted in most PWS patients contains multiple GABA_A_​R subunit genes (e.g., GABRB3, GABRA5, GABRG3), whose loss has been associated with altered receptor binding and neuropsychiatric symptoms [35, 36]. Although our necdin-specific model (Ndn + m/−p) does not involve deletion of these subunits, we observed intact GABA_A_​R α2 expression and phasic activity in the LC, suggesting that GABA_A_​R dysfunction may be more relevant to patients with larger deletions. In contrast, our findings consistently point to impaired GABA_B_R function as a central feature in necdin-deficient mice. These results are aligned with studies using transcranial magnetic stimulation (TMS) in individuals with PWS, which have reported suppressed intracortical facilitation (ICF)—a biomarker associated with altered GABA_B_ ​, but not GABA_A_, receptor function [37]. Thus, while GABA_A_​R alterations may vary based on genetic subtypes, GABA_B_R signaling deficits may represent a convergent mechanism across PWS etiologies.

Astrocytic GABA release has emerged as a key regulator of tonic inhibition in multiple brain regions, and its dysregulation has been implicated in several neurological and neurodevelopmental disorders [38]. In Huntington’s disease and Alzheimer’s disease models, aberrant astrocytic GABA release contributes to altered excitation–inhibition balance and cognitive deficits [38]. Moreover, GABA_B_ receptors expressed on glial cells modulate astrocyte morphogenesis and signaling, further linking glial GABAergic mechanisms to network function [39]. At the circuit level, excessive astrocytic GABA release in the ventral tegmental area has been shown to augment tonic inhibition of local GABAergic neurons, thereby disinhibiting dopaminergic projections to the nucleus accumbens and altering reward-related behaviors [40]. In PWS, necdin deficiency adds a unique dimension to these mechanisms. Fujimoto et al. demonstrated that necdin normally suppresses EGFR/ERK signaling in cortical progenitors, restraining astrocyte differentiation, and loss of necdin thus promotes astrocytic proliferation or altered maturation [28]. In line with this, our study found that while in vivo astrocyte counts near the LC remained unchanged, Ndn + m/−p astrocytes displayed accelerated proliferation in vitro and secreted more GABA at later developmental stages. Given the LC’s critical role in attention, arousal, and stress responses, persistent glial-driven inhibition may contribute to the neuropsychiatric manifestations of PWS, including compulsivity, emotional dysregulation, and attentional deficits. Together, these data support a model in which necdin deficiency disrupts both neuronal and astrocytic components of GABAergic signaling, creating a pathological tonic inhibitory tone that parallels glial mechanisms described in Alzheimer’s, Huntington’s disease, and autistic spectrum disorder [38]. 

Despite the strengths of our study, several limitations warrant discussion. First, the Ndn + m/−p mouse model captures only a subset of the genetic abnormalities found in typical PWS deletions and may not fully recapitulate the broader spectrum of GABAergic dysfunction. While our data support preserved GABA_A_R function in this model, this may not apply to individuals with deletions involving GABA_A_R subunit genes. Second, although we observed functional alterations in GABA_B_​R, we did not assess downstream intracellular pathways, receptor binding affinity, or post-receptor coupling efficiency. Third, the increased GABA secretion observed in cultured Ndn + m/−p astrocytes may not fully reflect in vivo dynamics, where astrocyte-neuron interactions are modulated by complex environmental cues. Lastly, behavioral correlations of LC GABA_B_​R dysfunction were not directly assessed in this study. Future investigations using in vivo manipulations of GABA_B_​R signaling, paired with behavioral assays, will be essential to establish causal links between LC hypoactivity and neuropsychiatric phenotypes in PWS.

## Conclusion

In conclusion, this study identifies selective impairment of GABA_B_R-mediated tonic inhibition in LC-NE neurons as a key neurophysiological abnormality in necdin-deficient mice. Complementary astrocyte studies revealed enhanced proliferation and increased GABA release in necdin-deficient cultures, suggesting that altered astrocytic regulation of ambient GABA may further exacerbate inhibitory imbalance in the LC microenvironment. Together, these findings point to a dual mechanism—loss of functional GABA_B_R signaling in neurons combined with excessive astrocytic GABA release—that may drive LC hypoactivity and contribute to the attentional, compulsive, and emotional dysregulation characteristic of Prader–Willi syndrome. Targeting GABA_B_R signaling pathways, along with modulation of astrocytic function, may provide novel therapeutic avenues for alleviating neuropsychiatric symptoms in PWS and related neurodevelopmental disorders.

## Data Availability

The raw data supporting the conclusions of this article will be made available by the authors without undue reservation.

## Abbreviations

ACSF: Artificial cerebrospinal fluid
AP: Action potential
CNS: Central nervous system
PWS: Prader-Willi syndrome
LC: *Locus coeruleus*
GABA: Gamma-aminobutyric acid
PB: Phosphate-buffer
SFR: Spontaneous firing rates
TH: Tyrosine hydroxylase
WT: Wild-type
